# Adaptive Temperature Compensation in Circadian Oscillations

**DOI:** 10.1371/journal.pcbi.1002585

**Published:** 2012-07-12

**Authors:** Paul François, Nicolas Despierre, Eric D. Siggia

**Affiliations:** 1 Ernest Rutherford Physics Building, McGill University, Montreal, Quebec, Canada; 2 Ecole Polytechnique (member of ParisTech), Palaiseau, France; 3 The Rockefeller University, New York, New York, United States of America; University of Warwick, United Kingdom

## Abstract

A temperature independent period and temperature entrainment are two defining features of circadian oscillators. A default model of distributed temperature compensation satisfies these basic facts yet is not easily reconciled with other properties of circadian clocks, such as many mutants with altered but temperature compensated periods. The default model also suggests that the shape of the circadian limit cycle and the associated phase response curves (PRC) will vary since the average concentrations of clock proteins change with temperature. We propose an alternative class of models where the twin properties of a fixed period and entrainment are structural and arise from an underlying adaptive system that buffers temperature changes. These models are distinguished by a PRC whose shape is temperature independent and orbits whose extrema are temperature independent. They are readily evolved by local, hill climbing, optimization of gene networks for a common quality measure of biological clocks, phase anticipation. Interestingly a standard realization of the Goodwin model for temperature compensation displays properties of adaptive rather than distributed temperature compensation.

## Author Summary

Circadian clocks are biological oscillators which evolved to couple the internal rhythm of animals, plants and even some bacteria to the alternation of light and day. Circadian oscillators are temperature compensated, i.e. they keep a 24-h period irrespective of the temperature of the organism. This is surprising, since many biochemical parameters, including average concentration of clock proteins, vary with temperature. From dynamical system theory, we therefore expect changes in both period and relative lengths of features in the phase response curve which are not seen. We couple mathematical modelling and computational evolution of gene networks to formulate a novel explanation for temperature compensation that accords better with experimental facts than alternatives. Our model has deep mathematical connections with the process of biochemical adaptation, by which cells respond to temporal gradients of signals rather than their absolute value.

## Introduction

It has long been recognized [1] that the defining characteristics of biological circadian clocks are (1) a free running period of order 24 hrs in the absence of any periodic stimulus, (2) entrainment by a periodic light-dark signal and (3) temperature compensation of the free running period. Equally important is entrainment by a 24 hour period temperature variation [2]–[6]. In a natural environment, temperature and light stimuli are correlated but laboratory experiments show a well defined response to each stimuli separately in non endothermic organisms. In mammals phase coordination between various organs is brought about by the normal circadian temperature oscillation [7]. Temperature might have been the original zeitgeber since most biochemical reactions respond to temperature [8] and few directly to light. Yet the response to temperature seems to impose two conflicting requirements on circadian oscillators, the period should be temperature independent yet there should be strong entrainment by temperature, i.e., a small amplitude signal elicits a full phase shift within one or two periods.

There is presently only very sparse data on how the principal steps that additively determine the 24 hr period vary with temperature [9]. A common supposition is that all biochemical rates vary randomly with temperature with a typical 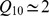 ie rates double for a 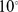
C temperature change, and evolution imposes a constraint on the individual Q values to keep the period constant [10]–[13]. However if the on/off times of various clock genes vary substantially with temperature, one might expect to see changes in the phase response curve to light, 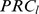. None were observed by Zimmerman et al. [14] which lead them to the strong assumption that the limit cycle orbit is temperature invariant, along with the period [15]. Temperature entrainment in their model occurs via an additional adaptive sensor variable that responds to temperature change but assumes a constant temperature independent value when the temperature is constant. However numerous measurements have shown that the mean message or protein levels of clock genes do vary substantially with temperature (e.g. [3], [5]) thus undercutting the appeal of their model. The shape of the PRC in response to either light or temperature is a phenotype of a circadian oscillator, and as such may be under selection. We want to explore more constrained models of circadian oscillators that are temperature entrainable and leave the shape of the PRC as well as the period temperature invariant. To do this we have to assume that the temperature only enters a few parameters in the model. The challenge is how to reconcile this assumption and more generally temperature compensation itself, with the many observations that show message and protein levels are very sensitive to temperature [16]. The ostensible conflict between variable biochemistry and an invariant period stems from the assumption that all rates separately contribute to the clock period. Models such as delayed negative feedback and relaxation oscillators certainly exist where the period depends on only a subset of the parameters ([17]). In addition, a single model parameter such as a degradation rate may depend on multiple biochemical events that when combined are temperature independent.

Sensory adaptation is an apt analogy for how the clock period becomes temperature invariant yet temperature entrainable, and forms the mathematical basis for our model. Assume the temperature is the ‘stimulus’ and it only enters the model in a few specific terms consistent with adaptation to the stimulus. Then as we will show the period and PRC shape are temperature independent. The response of an adaptive system to the *derivative* of the stimulus ensures entrainment by temperature oscillations. Some other variables in the adaptive system buffer the stimulus and vary systematically with temperature which eliminates a flaw in the Zimmerman et al. adaptive model.

The temperature response of this new class of so called *adaptive* models is structural and resides in how the temperature enters the network, namely in select terms. Adaptive circadian models are not contrived. Both their topology and parameters are easily evolved by incremental improvements in a commonly assumed fitness namely linear correlation with a noisy periodic training signal. When derived this way, our circadian models display a shape invariant PRC and the property of temperature compensation is robust to a 2× change in most parameters in the model. The period may change with parameters but it will remain temperature independent.

We begin with a summary of experimental facts that cast doubt on the literal application of a distributed temperature compensation to circadian clocks and then introduce a series of models of increasing complexity and realism based on the idea of adaptive compensation. The Discussion enumerates some surprising experimental consequences of adaptive temperature compensation.

### Models of temperature compensation

The most prevalent model of temperature compensation is also the most parsimonious in that it makes no structural assumptions about how temperature enters the network equations, and was proposed by Ruoff and Rensing [10]. The period, 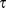, depends in an unknown way on all the constants in the model e.g., the rates and equilibrium constants 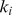 in a Michaelis-Menten description. Their temperature, T, dependence can be expressed in Arrhenius form, 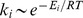. Then temperature compensation is expressed as
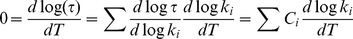
(1)and becomes a linear constraint on the 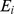 since the 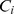 are supposed constant over a physiological temperature range. Since Eq 1 imposes a global constraint on all parameters, we describe it as “distributed” temperature compensation.

This model though parsimonious is not intuitively satisfactory in all respects, though it does not directly contradict any experiment. As noticed by Tyson and coworkers, several mutants do not appear fully consistent with distributed compensation [18]: in particular, more than 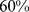 of mutants in fly and Neurospora whose period is different from 24 hrs retain compensation (see [18] Table 1 and references within and [19], [20]). So we have to assume there are genes that affect the period, yet are not temperature dependent i.e., are compensated locally, or do not change their temperature dependence when mutated. Also model parameters can subsume multiple biochemical events that collectively appear temperature independent [21]. Of course there has to be temperature dependence somewhere to allow entrainment.

Other fly mutants like 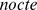 have a temperature compensated period but fail to entrain [4]. This is unexpected if several terms are temperature dependent; from our understanding of typical non linear oscillating systems any coupling to temperature will lead to entrainment. Finally if multiple Michaelis-Menten constants vary with temperature one would expect the shape of the orbit to vary in an arbitrary way with temperature. If the orbits change, the phase response curves to either light or temperature should too, since they just measure the isochrons (surfaces of constant phase) around the orbit.

## Results

### Temperature compensation as adaptation

**Figure 1:**
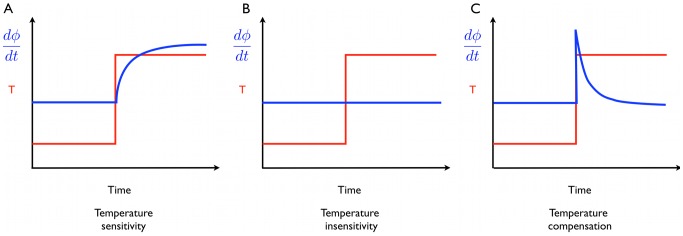
Three idealized behaviours in response to a temperature step. Red is temperature, blue is 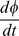 for the clock phase. (A) Temperature sensitivity. The clock ticking rate (and consequently its period) changes with temperature. (B) Temperature insensitivity. Nothing changes with temperature. (C) Temperature compensation. The clock runs faster just after a temperature step (explaining entrainment and phase shifts) but then returns to its initial value, so that period of the clock does not change.

The dual properties of a temperature independent period and strong entrainment by an oscillating temperature are tantamount to asserting that the time rate of change of the phase (angular velocity) around the orbit is adaptive, Fig. 1. Adaption means that subject to a temperature step, the system responds with a pulse but then returns to the same steady value it had before the step (see for instance [22]). A minimal expression of this idea, now for the phase, is given by an idealization of the situation envisioned in [14]

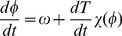
(2)where 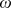 is the angular frequency of the oscillator (period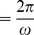) and the temperature is 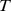. The angular velocity only deviates from its temperature independent value when the temperature changes.

A temperature step 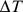 applied when the oscillator has phase 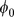 induces a phase shift 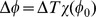. If we compute the cumulative effect of a rapid step-up step-down in the temperature one finds a total phase change:
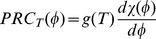
(3)which is the definition of the PRC with respect to temperature changes and where 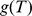 depends on details of the temperature pulse (e.g., duration and intensity). Thus the PRC at different temperatures can all be superimposed by scaling the overall amplitude 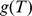; they have the same shape [14]. The magnitude of 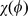 and its 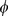 dependence both control how well the oscillator is entrained by a periodic temperature signal [23]. Whereas 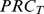 is proportional to the temperature pulse no matter how large, in more realistic models the phase response to a light pulse occurs through the degradation of one or more clock components (like TIM in fly [24]) and clearly saturates. Since there is no basis in our models for designating a light sensitive variable, we define a 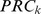 with respect to any parameter 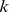 by making a rapid excursion in the parameter from its nominal value and back to baseline.

While Eq. 2 may seem very artificial, we show next that its principal features are recovered in a widely used model for temperature compensation in the *Neurospora* clock.

### Temperature compensation in the Goodwin model rescales the limit cycle

Ruoff and coworkers have used the Goodwin model [10] as a generic negative feedback oscillator with which to model the circadian clock in Neurospora.
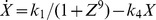
(4)

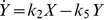
(5)

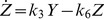
(6)


Following [11] X would roughly correspond to FRQ RNA, Y for cytoplasmic FRQ and Z to nuclear FRQ. For simplicity, in the following, we will call 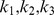 production rates and 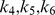 degradation rates.

If we assume that variable 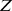 is larger than 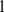 (as it actually is in ref [10]), we can neglect 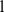 relative to 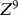 in the first term of Eq. 4. One can then rewrite the equations for rescaled variables, 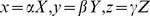, so as to reduce Eqs. 4–6 to
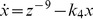
(7)

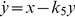
(8)

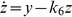
(9)using 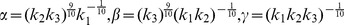. So the production rate parameters have been completely absorbed in the rescaling and the degradation terms are not affected. This has several consequences (Fig. 1):

- the amplitude of the orbit varies with the production rates, while the period is independent of them.
- the oscillator orbit undergoes a linear transformation after a temperature step if only the production rates are temperature dependent i.e., 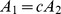 where 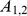 are the orbit coordinates at two temperatures and the rescaling factor 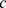 is temperature dependent.
- the phase response curve, defined by multiplying one or more coefficients by a time dependent factor, is invariant under any constant rescaling of the production terms, since the transformation from Eqs. 4–6 to 7–9 clearly applies with the temporally modulated coefficients.

**Figure 2:**
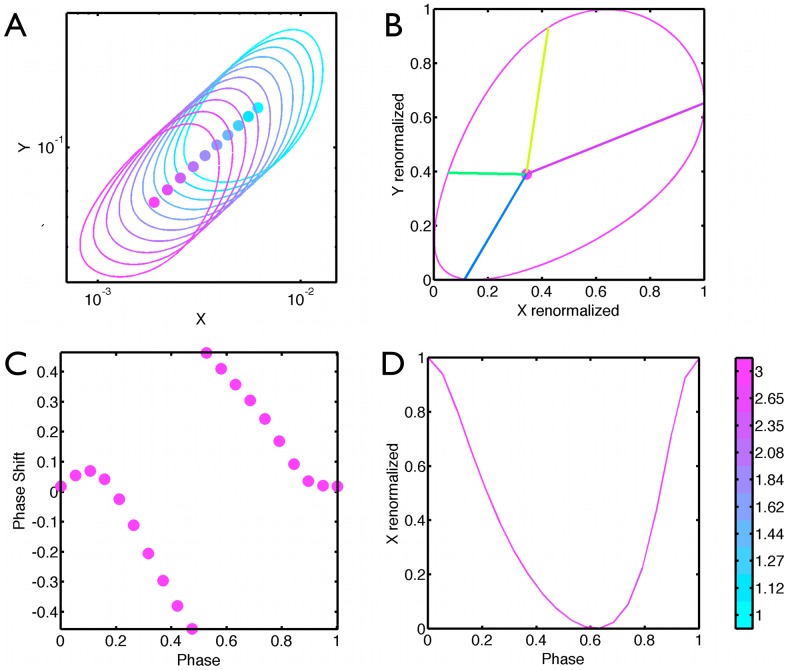
Scaling of the limit cycle for the Goodwin model. The parameters are 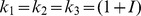, 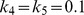, 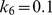, where 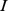 is the input, a proxy for temperature. (A) limit cycle for different values of the input in XY space. (B) linear rescaling of the orbits to [0,1] collapses them all onto a single orbit. The central dots indicate the unstable (Hopf) fixed point. Straight lines from the fixed point separate the cycle in 4 periods of equal duration. (C) PRC of the Goodwin model for different input values is invariant. PRC was computed by adding a degradation term of 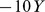 in Eq. 5 for 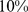 of the period. (D) Variable X as a function of phase for the limit cycles at different temperatures, scaled as in B. The maximum of X is defines phase 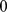 for the PRC. There is perfect overlap for different input values (as shown on the scale bar for all panels) as expected.

These remarks then explain the results of Ruoff and coworkers [10], [11] on temperature compensation in the Goodwin model, since they chose small activation energies for degradation rates, and large ones for the production rates. Thus the amplitude of the clock changes substantially with temperature, while the period is fixed since the degradation rates were not changed. The Goodwin model for their parameters is not an example of distributed temperature compensation as sometimes claimed, but rather is effectively temperature independent! Thus the PRC shape is also temperature independent Fig. 2D.

### Evolving temperature compensated oscillators

The linear transformation on the orbits induced by temperature and the temperature invariant PRC we derived from Eq. 7–9 seems very specific to the Goodwin model, and we would like to demonstrate that the same properties are found in a wider class of models. As explained above, temperature compensation looks formally very similar to biochemical adaptation. Thus it is natural to ask if we can build temperature compensation upon an adaptive network for temperature. To be consistent with mutants such as 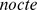, we are looking for models where temperature *explicitly* changes only very few parameters : temperature compensation in this limit is expected to rely on structural properties rather than the distributed compensation mechanism. Given the complexity of these constraints, we use *in silico* evolution as a mathematical tool to generate temperature compensated models.

Our simulations evolve both the gene network and the parameters as we have done previously [22], [25]–[27], and we allow just transcription and protein-protein interactions, PPI, (see Supplementary Text S1 for more details). Temperature is introduced through a so called input variable, 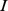,which typically couples to just one or a few other variables. The input will vary over a range of 2 or more to represent a substantial temperature dependence as defined by a typical 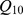 parameter.

To emphasize the connection to adaptation we initialize our simulations with a simple two gene adaptive network, shown in 3A, that we evolved previously [22] and is standard [28]:
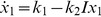
(10)

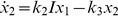
(11)(We hence forth generically use lower case variables in all equations with no implication that they are rescaled in some manner.) The identical temperature dependence is implied wherever the parameter 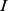 occurs in the equations. Thus in Eqs. 10, 11, 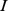 controls the rate of an interaction that consumes 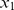 and makes 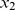, so there is really only one instance of 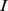. Adaption is realized by the output, 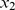 that responds to a temperature step with a pulse (as in Fig. 1 C) but ultimately returns to the value 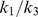. The absolute level of temperature is reflected in 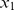.

In contrast with the model of Zimmerman et al. [14] or Eq. 2, where temperature was filtered through an adaptive system and only the output, essentially 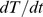, was coupled to the clock variables, this adaptive initial system forms the core clock components. This ensures that the mean levels of some variables analogous to 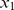 are required to vary substantially with temperature as is observed in natural systems. When we evolve a temperature compensated circadian oscillator, the objective function has to overcome the tendency for all features of the system to vary with Input, our surrogate for temperature.

The evolution optimizes the *fitness*, F, defined here as a sum of two functions. The first part of the fitness, 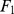 is average correlation between the output, 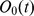 (the model variable that evolves from 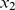 in the adaptive system), and an Input 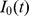. Let brackets denote the average over the time window for the fitness evaluation, typically 12 periods of the input and 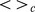 the subtracted normalized correlator i.e., 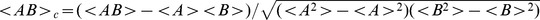. Then:
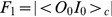
(12)We take 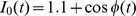, where 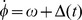, 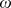 defines the period. There are one or more random jumps in the phase defined by 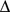 for 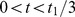 : the phase jumps favor entrainment since the fitness forces the output to follow the jump.

The second part of the fitness 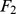 is the average correlation between output 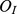 - entrained by a different Input signal 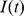 - and output 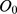 computed in the first part of the fitness
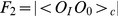
(13)


**Figure 3:**
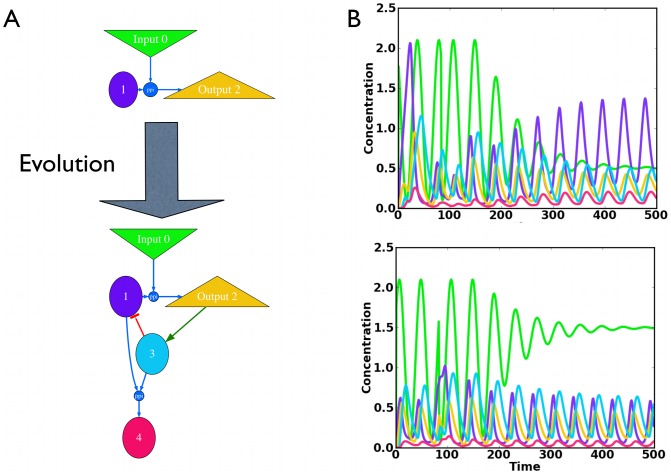
Evolution of adaptive temperature compensation. (A) Sketch of the initial adaptive topology and its subsequent network evolution. Parameters and equations are given in Supplementary Text S1. Properties of this network are further detailed in Figure 4. The input, 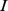 which models the temperature; the output 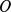 which adapts; and 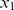 (Eqs 10 11) changes substantially with a temperature step and functions as a buffer are color coded. (B) Temporal behaviour of the evolved network for two different input trajectories (the colors follow (A)). The input oscillates, undergoes a random phase shift around time 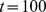, and decays exponentially to a constant value. Note only the mean of the buffer 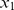 changes substantially with the terminal input value.

For the first third of the integration period, 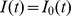, then 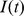 tapers down a constant for the remainder of the integration, as shown in Fig. 3B. The constant input for 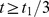 encourages an autonomous oscillator (rather than a system that merely follows the initial input oscillations) and its variable level directly enforces temperature compensation. As can be seen, Fig. 3B, the mean of 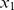 continues to register the final input level, as it does in Eq. 10, even when oscillating, while the amplitude of the other variables is nearly independent of input level (i.e., our temperature surrogate). The correlation function 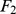 between Outputs computed for different constant Input values ensures that the shape of the limit cycle for the different terminal Input values is the same. 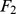 is computed for different terminal values of the Input between 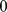 and 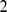, then averaged. The final fitness is 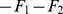, which when minimized ensures that the Output is fully correlated with an oscillating Input, and that Output for different Inputs constant values behave in similar way.

One of the simplest models found by numerical evolution is presented in Fig. 3A. The first step in evolution adds variable 3 which creates a delayed negative feedback from the output back to itself by repressing 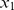 and creates an oscillator. The PPI between 1 and 3 is added next and actually improves the temperature compensation illustrated in Fig. 3B.

**Figure 4:**
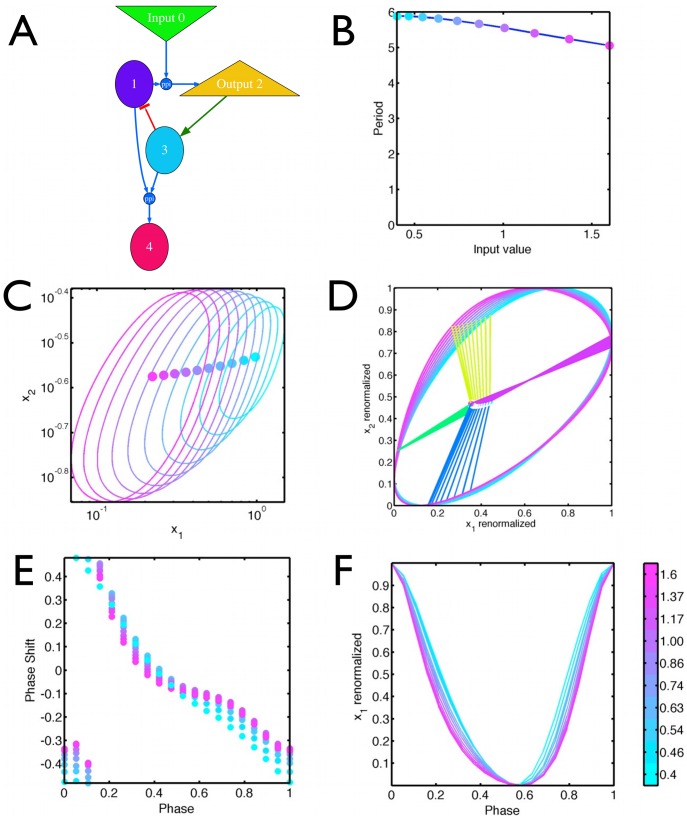
Scaling of the limit cycle for first evolved model. (A) Sketch of the model. Parameters and equations are given in Supplementary Text S1 (B) Variation of the period with input level demonstrating compensation. (C) limit cycle for different values of the input in 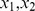 space. Limit cycle varies over almost one order of magnitude in 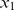 while the period changes by 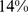. The input values follow the color bar in F. (D) Rescaling the limit cycles to the unit interval in each variable shows almost perfect collapse for different input values. Circles indicate the fixed point. (E) PRC for different input values, represented by different colors. The PRC was computed by adding a degradation term of 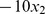 for 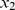 for 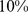 of the period. (F) 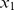 as a function of phase for the limit cycle at different temperatures. The maximum of 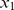 is defined as phase 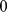 for the PRC. There is almost perfect overlap. Panels D–F here and the following figure, demonstrate our contention that the evolved models replicate essential properties of the Goodwin model even though there is no direct parameter rescaling.

Schematically, compensation in this model works in a way very reminiscent to biochemical adaptation in the network used to initialize evolution: variable 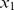 buffers most variation by essentially scaling as 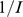 (Figs. 3 and 4, Supplementary Table S1) while other variables vary much less in comparison. The reason is that the effective reaction rate controlling 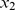 is 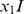 and is therefore roughly Input invariant, and consequently so is the shape of the limit cycle.

The properties of the oscillations defined by the network in Fig. 3 as a function of the input level are shown in Fig. 4. Oscillations arise as a Hopf bifurcation and persist for input values in the interval 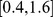. There is no change in period for two inputs that cause a 2× change in the mean of 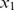 and only a 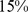 change in period when 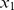 changes by its maximum possible range of 5×. (Fig. 4B and C, Supplementary Table S1). These values are perfectly compatible with the typical variation in clock period with temperature : for instance, in zebrafish, the oscillation amplitude and average value of 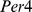 changes roughly by 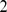 and period decreases by 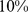 for a temperature change from 20 to 30 C [5]. In Neurospora, the period decreases by 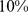 between 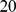 and 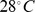 for the control strain KAJ10 [3], [29]. In a pure WT background, the average concentrations of FRQ protein are roughly multiplied by 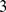 over the same range while the period decreases of around 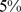 [11], [30], and at higher temperature, a 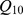 of 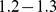 is observed [30].

Clocks built from an adaptive system, share a feature of the Goodwin model that the orbits for different inputs, as well as the location of the unstable Hopf fixed point, can be superimposed by a linear rescaling, Fig. 4D. Thus the phase corresponding to the limits of the orbits is temperature invariant. A stronger form of this property is seen in the PRC. If we simulate a PRC by zeroing the output at a defined phase, Fig. 4E, we see that they too collapse for all inputs even though we are administering a strong perturbation.

We verified numerically that the PRC are shape invariant whether derived from a strong localized decay rate applied to any of the adapted variables in Fig. 3 (i.e., variables other than 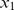), or by momentarily jumping up the production rates of adapted genes (see Supplementary Fig. S1). (The PRC defined for the decay of the buffer variable 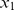 is less well conserved since its more mixed in with a change in input level)

Since the fitness is linear correlation with a sinusoidal reference phase, it is maximum when the solution is itself sinusoidal and optimally remains so when the temperature is shifted, thus explaining the linear covariance of the orbits with temperature. In general the evolved models behave as if they were near the Hopf bifurcation, yet do so over a parameter range that causes a 10× change in the orbits.

We have also verified that a two-fold variation in parameters does not appreciably degrade the period compensation shown in Fig. 4B (Supplementary Fig. S2), i.e., the period changes but it remains temperature independent. However doubling the PPI between the input and 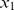 is equivalent to doubling the input range and thus shows more period variation since its like doubling the temperature range.

Thus parameters are not tuned, and their general magnitudes are easy to find by a simple local hill climbing algorithm (a.k.a. gradient search). Two other evolved networks with similar properties are presented in Supplementary Figs. S3 and S4.

### Temperature compensation via feed-forward adaptive networks

Network of Fig. 4 is adaptive via a feedback mechanism, however feed-forward networks form the other main class of adaptive networks and their evolution into oscillators gives rise to the Mixed Feedback Loop (MFL) that is common in circadian clocks [22], [28] and has been proposed by one of us as a core model for the *Neurospora* circadian clock [31], [32]. The MFL is an oscillator in which a transcriptional activator A activates a gene B and then A and B dimerize. Examples include WCC and FRQ in *Neurospora*, Clock and PER/TIM in fly, Clock/BMAL and PER/CRY in mammals. If the production rates of A and B (via transcription or translation) depend in a similar way of the input, it can be shown that the fixed point for A is adaptive as we evolved in [22]. Strikingly, for this adaptive MFL, the limit cycle shape and period are then automatically independent of the input value and still entrain, as seen in Fig. 4 and shown analytically in the Supplementary Text S1. This is again a structural property of the network which can be understood mathematically : the strong PPI makes the system function as a relaxational oscillator between two states either A or B high. The fact the production rates of both genes have similar dependence on the input then implies that the input dependence can be scaled out of the equations, in analogy to the Goodwin model, and the period is input independent.

### Evolving variable PRCs and temperature compensation

We further wondered if computational evolution is able to select for different categories of compensated clocks, where the limit cycle and PRCs depend much more significantly on the input. We modified the fitness so it continued to favor entrainment to temperature 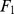, but we dropped the linear correlation 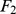 so as not to constrain the shape of the output for the free running clock. Instead, we computed the number of peaks of the Outputs for different constant Input values and forced it to be equal to the number of peaks of 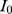 : this ensures that only the period of the Output is constrained, but not the shape of the limit cycle.

**Figure 5:**
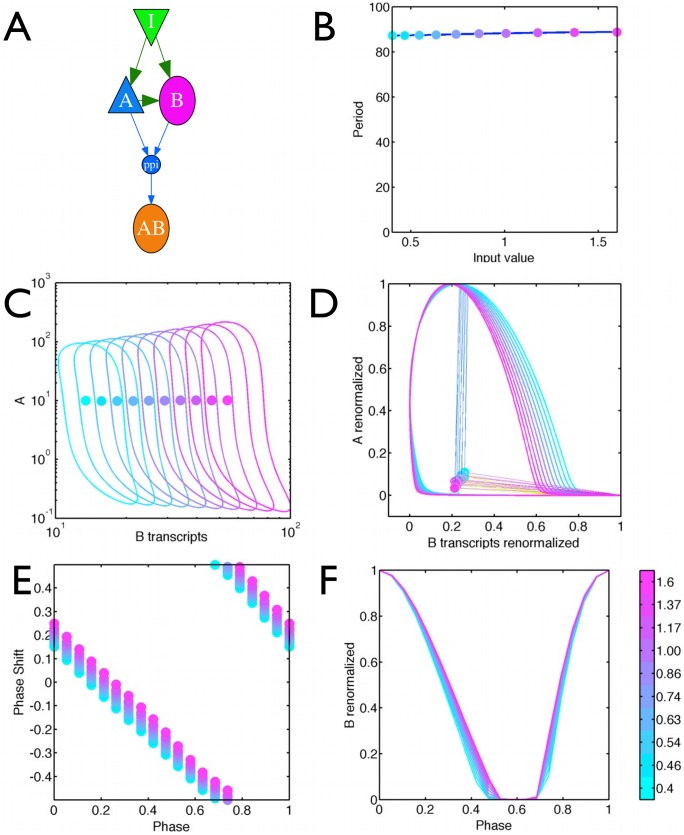
Scaling of the limit cycle for the Mixed Feedback Loop adaptive model. (A) Sketch of the model. Parameters and equations are given in Supplementary Text S1 (B) Variation of the period as a function of input values. (C) limit cycle for different values of the input in the B-transcript, A-protein plane. Limit cycle varies over almost one order of magnitude in B-transcript level, while the period changes by a few percent. Note that the fixed point for A is adaptive (independent of input). (D) Linear rescaling of the limit cycles to the unit interval in each variable showing almost perfect collapse for different input values. Circles indicate the fixed point. Color code follows bar in panel F (E) PRC for different input values, represented by different colors. The PRC was computed by adding a degradation term of 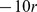 for B transcripts for 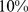 of the period (see equations in Supplementary Text S1). (F) B as a function of phase for the limit cycle at different temperatures. The maximum of B is defined as phase 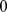 for the PRC. There is almost perfect overlap.

Properties of a network evolved under this scheme is described in Fig. 5. This network obviously is much more complex, with two interconnected transcriptional negative feedbacks explaining oscillations (via species 4 and 7). The Input enters in various places, not only in the imposed original core network (species 1 and 2) but it also acts independently on species within the two negative feedback loops (species 5 and 4). This system is therefore closer to the traditional picture of distributed temperature compensation, with the input entering the equations at various places.

**Figure 6:**
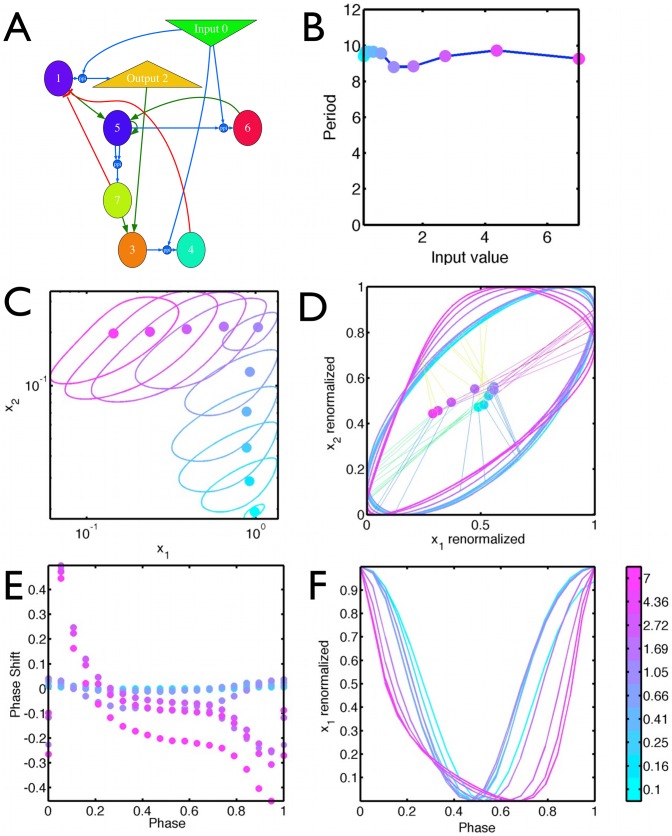
Absence of scaling for an evolved model with distributed temperature compensation. (A) Sketch of the model. Parameters and equations are given in Supplementary Text S1 (B) Variation of the period as a function of input. (C) limit cycle for different values of the input in 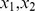 space. Limit cycle varies over one order of magnitude in variable 1 and 2 while the period changes at most 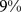 (D) Rescaling of those limit cycle to the unit interval for each variable. The orbits for different inputs no longer scale. Circles mark the fixed point (E)The PRC for different input values do not scale. The PRC was computed by adding a degradation term of 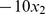 for variable 2 for 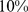 of the period. (F) Variable 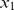 as a function of phase for the limit cycles at different temperature. Its rescaled maximum of 1 is defined as phase 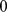 for the PRC.

This network displays autonomous oscillations for input values higher than 0.1. Remarkably, while the input is changing from 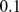 to7, variables 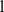 and 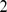 vary themselves over more than one order of magnitude (Fig. 6 C), while the period only varies by about 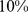 (Fig. 6B). Clearly, neither limit cycle shapes nor PRCs are conserved over this input interval (Fig. 6 C and D). This illustrates our contention in the Introduction that distributed temperature compensation is incompatible with a shape invariant PRC. Unexpectedly, both PRCs and limit cycle shapes cluster onto two different regimes,namely inputs above and below 0.7, and in each regime the scaling has all the characteristics of the adaptive compensation in Fig. 4.

## Discussion

We have exhibited a sequence of *adaptive* models for temperature compensation and entrainment in circadian oscillators. They arise naturally when a system that adapts to temperature steps evolves to become an oscillator. These models can be generated by a gradient search or hill climbing optimization, thus there are no subtle correlated changes that have to be made to generate these models. Prior analysis of temperature compensation e.g., [12], [13], has focused on distributive models. An exception is [18], which however does not temperature entrain since the period is given by model parameters.

Properties of these models (beyond the temperature compensation and entrainment that we imposed on the evolution) are:

1. temperature *explicitly* enters the network in a limited number of terms,
2. clock components oscillate around means that either are temperature independent (and are coupled to the adaptive variables) OR vary and buffer the temperature change (e.g., variables 2,3 vs 1 in Fig. 4),
3. orbits rescale linearly with temperature, and in addition the phases that define extrema on the orbits are invariant,
4. the shape of the PRC is temperature independent, when defined by an augmented decay rate on the adapted variables.

Experiments from a variety of organisms are better explained by adaptive rather than distributive temperature compensation. In fly, mutations in *nocte* abolish temperature entrainment but not compensation [4]. Since periodic modulation of any parameter should generically (i.e., other than for special choices of parameters) entrain a nonlinear oscillator, the *nocte* mutant is very suggestive of item (1). The numerous mutants with altered periods that continue to temperature compensate [18] suggest that the net result of the biochemistry that defines the transitions between the principal phases of the clock is temperature invariant. There is contradictory data about the loss-of- function mutation *cryb* and temperature. Reference [4] notes *cryb* flies still temperature entrain, but [33] show the temperature PRC is almost flat.

In saturating light the fly PRC are temperature invariant [14] and the activity peak in light-dark synchronized flies is temperature independent as first observed by Pittendrigh [8] which support items (3–4). Adaptive compensation in fly would also suggest that clock phases defining the interval of *tim* expression and its maximum are temperature invariant [34] (item 3)

In cyanobacteria circadian clock temperature compensation occurs through the KaiC component alone and temperature compensation persists in mutants with periods substantially different from 24 hrs [35], suggesting again localized temperature compensation. Importantly, KaiC ATPase rate is temperature independent and obviously does not follow an Arrhenius law [35]. Evidence for an adaptive mechanism of temperature response as in Eq.2 where the temperature jump generated the phase shift, was provided in [2] (their ‘nonparameteric’ model).

In *Neurospora* the mean of the oscillating FRQ protein varies substantially with temperature and provides a mechanism for how a step up in temperature resets the phase [29]. The majority of circadian temperature effects seem to be mediated by FRQ [29], [36], supporting item (1).We consider FRQ analogous to our buffer variable 1 in Fig. 4. The mean of *frq* transcripts appears much less temperature dependent, supporting item (2). Data from [29] are consistent with the idea that phases of FRQ peaks do not vary much with temperature (item 3). The VIVID protein [37] is implicated in the temperature invariance of the PRC.

The situation appears less clear to us in plants, perhaps because there are many more duplicated genes in *Arabidopsis*. It has been suggested that two cycles could co-exist, one sensitive to temperature, the other sensitive to light [38] which is consistent with (1–2).

For all models presented here, properties 1–4, when they apply, are structural : for the Goodwin model this is due to the specific forms of the equation that allowed rescaling, in the MFL model the properties derive from the specifics of the coupling to inputs, and for Fig. 4 we verified 1–4 survive parameter variation. These properties would be difficult to understand unless temperature appeared in only a few terms of the equations.

Experiments that would most readily substantiate an adaptive model for temperature would be comprehensive data on the zeitgeber time of the maxima and minima of the clock components as a function of temperature. We predict their invariance, while a generic model of distributed compensation would predict that they move with temperature but of course continue collectively add up to the invariant period length. Temperature invariance of the extrema in the clock gene orbits, would suggest some degree of shape invariance in the PRC, but the later is in principal a separate prediction. The linear rescaling of orbits at different temperatures that we found in our models could be probed by time lapse imaging two out of phase clock genes. However the effect might not occur for all choices of genes if there was some saturation. In that situation the phases of extrema will be invariant and thus provide a more robust prediction.

The primary 24 h periodic pacemaker in nature is light. It is worth stressing that adaptation for light inputs themselves has been suggested in Neurospora, a phenomenon called photoadaptation [39], [40]. In 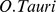, a computational study showed that phase shifts happen only when luminosity strongly changes [41], and this observation has been related to robust entrainment for all species [23]. These examples actually suggest a generic adaptive model for light sensing, just like described in Fig. 1C. Temperature variations are certainly correlated to sunlight [34], as well as other metabolic properties (such as the ADP/ATP ratio zeitgeber for cyanobacteria [42]) and could have been used as the original pacemaker. However, intrinsic day-to-day variations in the level of any zeitgebers would favor evolution of mechanisms to buffer these changes, and hence adaptation.

We have no definitive proposal for how almost all the temperature dependent biochemical rates disappear from the schematic or phenomenological models we are proposing for the circadian clock. We speculate that the shape of the PRC is under strong selection to remain temperature independent along with the period, and thus forces local compensation to render most model parameters temperature invariant, but leaving behind adaptive temperature dependence to allow temperature entrainment. The experimental implications of phase orbits that linearly rescale with temperature are sufficiently dramatic that their observation would render adaptive circadian models plausible though still surprising from the biochemical vantage point.

## Methods

For evolutionary simulations we follow [22] and use only transcriptional interactions and protein-protein interactions. Regulation of transcription of a protein B is modelled as a combination of Hill functions. Assuming that transcription factors 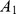 and 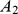 activate expression of gene 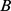 and that repressor 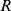 represses it, equation for 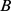 would then be:
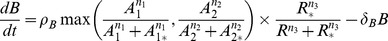
(14)

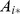
 and 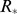 are threshold concentrations in Hill functions, 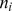 are Hill coefficients accounting for cooperativity. Parameters are chosen and evolved randomly. Equation 14 expresses that we assume an “OR” combinatorial between activators (i.e. one single activator is enough to activate trannscription) while repressors act multiplicatively.

Protein-protein interaction (PPI) are explicitly modelled using standard mass-action laws. For instance, if proteins A and B form a dimer C, the equations are:
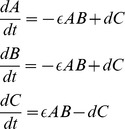



The fitness is computed for a population of networks, typically 40 in number. The most fit half of the population is retained, and a copy of each network is mutated and added back to the population to maintain its number. Parameter changing mutations are typically ten times as likely as topology changing events. Mutations are sampled according to their intrinsic rates and the generation time is chosen such that approximately one mutation occurs per network.

## Supporting Information

Figure S1
**Families of PRCs for model of** **Fig. 4** **.** All PRCs are computed by imposing a perturbation for 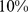 of the period of a cycle. (A) Strong degradation of species 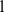 (as described in main text) (B) Strong degradation of species 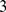 (C) Strong degradation of species 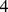 (D) 4× increase of transcription of gene 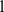 (E) 4× increase of transcription of gene 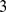 (F) 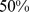 relative increase of Input. The strongest departure from input (temperature) invariance is in A since adding degradation to species 1 breaks the adaptation in the initial adaptive system composed of species 0,1,2 in Fig. 3.(PDF)

Figure S2
**Relative period as a function of Input for simulated mutants of networks in** **Fig. 4** **.** Period 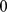 corresponds to absence of oscillation, periods are computed relative to the original network for the reference Input value of 0.4. Individual period for each mutant can be different from the original network, but for most mutants taken individually, period variation is comparable to the original network. (Left) Parameters individually divided by 2. The most significant relative difference (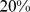) is for the parameter in dark blue which corresponds to the degradation rate of the Output (species 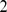). (Right) Parameters individually multiplied by 2. The most significant relative difference (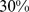) is for the parameter in yellow which corresponds to the coupling between the input and the network, so effectively multiplies the input range by 2.(PDF)

Figure S3
**A scaling model evolved with Fitness A.** (A) Sketch of the model. (B) Variation of the period as a function of Input. (C) Left : limit cycle for different values of the Input in 1–2 space. Limit cycle varies by a factor 4 for variable while the period changes by at most 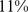 Right: Rescaling of the limit cycles to the unit interval for each variable. The orbits again collapse well. Circles mark the fixed point (D) Left : The PRC was computed by adding a degradation term of 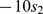 for variable 2 for 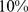 of the period. (Right) Variable 1 as a function of phase for the limit cycles at different temperatures. Maximum of 1 is defined as phase 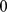 for the PRC.(PDF)

Figure S4
**A scaling model evolved with Fitness B.** (A) Sketch of the model. (B) Variation of the period as a function of Input. (C) Left : limit cycle for different values of the Input in 1–2 space. Limit cycle varies over one order of magnitude in variable 1 while the period relatively changes of at most 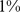 Right: Rescaling of those limit cycle to the unit interval for each variable. The orbits overlap again. Circles mark the fixed point (D) Left : Scaling of the PRC was computed by adding a degradation term of 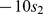 for variable 2 for 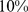 of the period. (Right) Variable 1 as a function of phase for the limit cycles at different temperature. Maximum of 1 is defined as phase 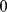 for the PRC.(PDF)

Table S1 **Detailed properties of limit cycle of network from** **Fig. 4** **for different Input values.**
(PDF)

Text S1 **Summary of computational evolution algorithm, detailed properties of network of** **Fig. 4** **, analytic derivation for the adaptive MFL network and equations for the different networks.**
(PDF)
