# Supplementary figures and images for: Adaptive Temperature Compensation in Circadian Oscillations
Source: PLoS Comput Biol. 2012 Jul 12;8(7):e1002585. doi: 10.1371/journal.pcbi.1002585 (PMC3395600; doi:10.1371/journal.pcbi.1002585)

A

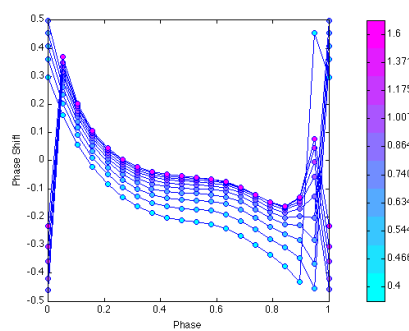

B

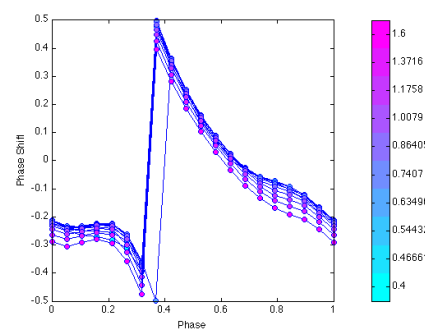

C

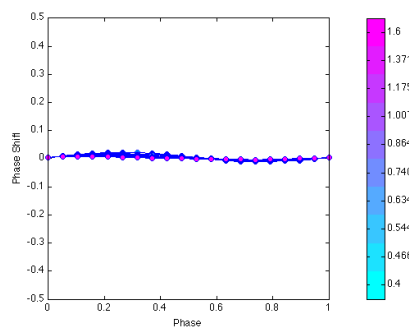

D

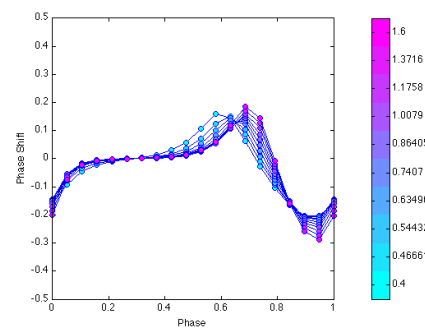

E

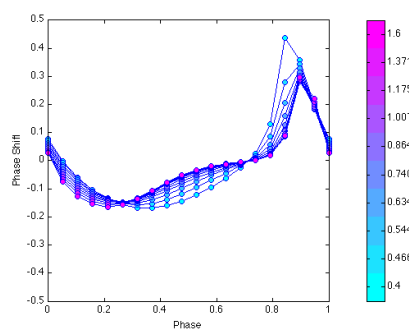

F

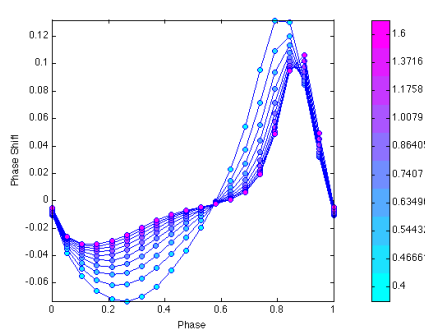

Supplement: Figure S1 — Families of PRCs for model of Fig. 4 . All PRCs are computed by imposing a perturbation for of the period of a cycle. (A) Strong degradation of species (as described in main text) (B) Strong degradation of species (C) Strong degradation of species (D) 4× increase of transcription of gene (E) 4× increase of transcription of gene (F) relative increase of Input. The strongest departure from input (temperature) invariance is in A since adding degradation to species 1 breaks the adaptation in the initial adaptive system composed of species 0,1,2 in Fig. 3. (PDF) [file pcbi.1002585.s001.pdf]

Division by 2

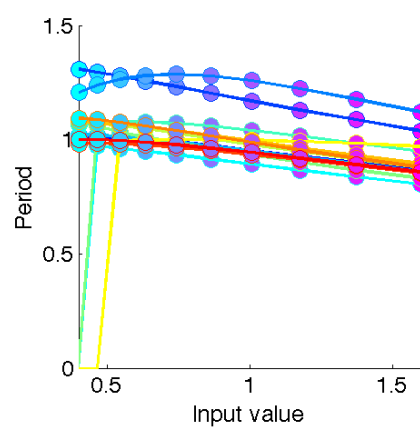

Multiplication by 2

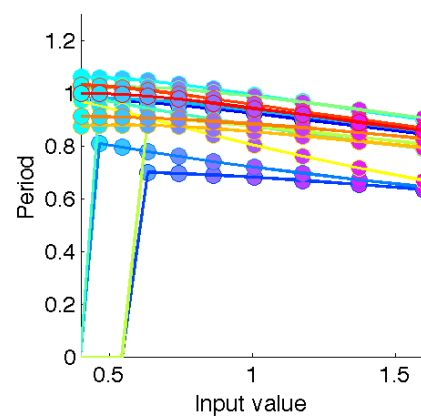

Supplement: Figure S2 — Relative period as a function of Input for simulated mutants of networks in Fig. 4 . Period corresponds to absence of oscillation, periods are computed relative to the original network for the reference Input value of 0.4. Individual period for each mutant can be different from the original network, but for most mutants taken individually, period variation is comparable to the original network. (Left) Parameters individually divided by 2. The most significant relative difference () is for the parameter in dark blue which corresponds to the degradation rate of the Output (species ). (Right) Parameters individually multiplied by 2. The most significant relative difference () is for the parameter in yellow which corresponds to the coupling between the input and the network, so effectively multiplies the input range by 2. (PDF) [file pcbi.1002585.s002.pdf]

**A**

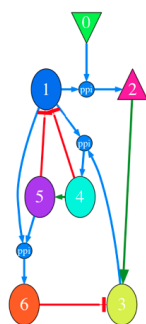

**B**

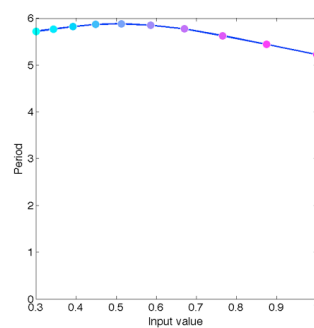

**C**

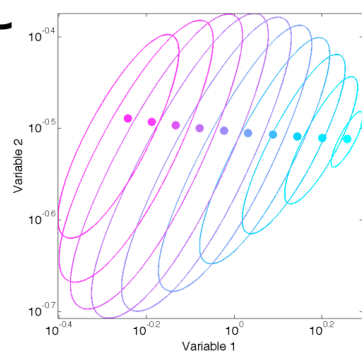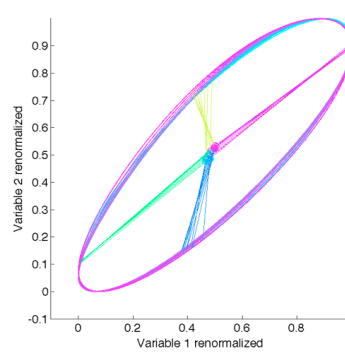

**D**

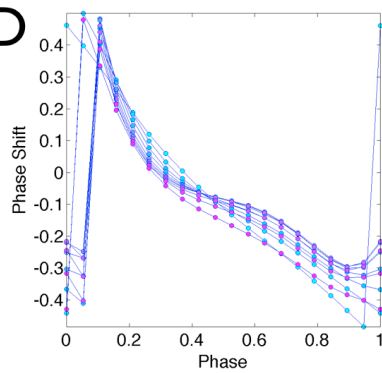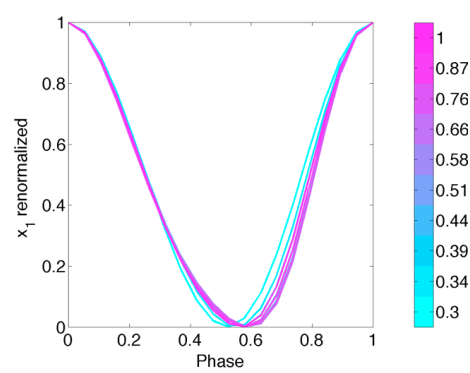

Supplement: Figure S3 — A scaling model evolved with Fitness A. (A) Sketch of the model. (B) Variation of the period as a function of Input. (C) Left : limit cycle for different values of the Input in 1–2 space. Limit cycle varies by a factor 4 for variable while the period changes by at most Right: Rescaling of the limit cycles to the unit interval for each variable. The orbits again collapse well. Circles mark the fixed point (D) Left : The PRC was computed by adding a degradation term of for variable 2 for of the period. (Right) Variable 1 as a function of phase for the limit cycles at different temperatures. Maximum of 1 is defined as phase for the PRC. (PDF) [file pcbi.1002585.s003.pdf]

**A**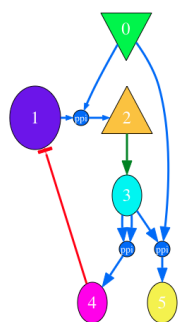**B**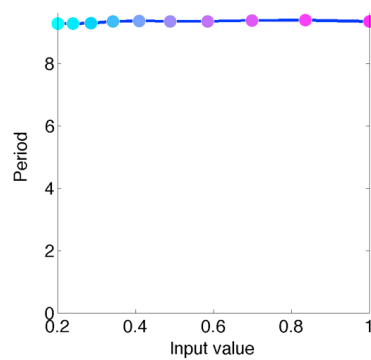**C**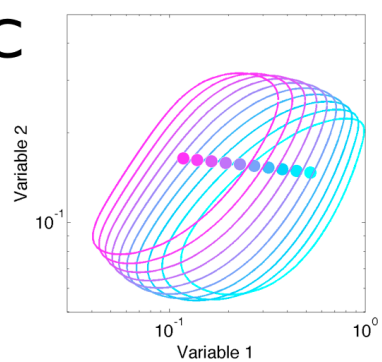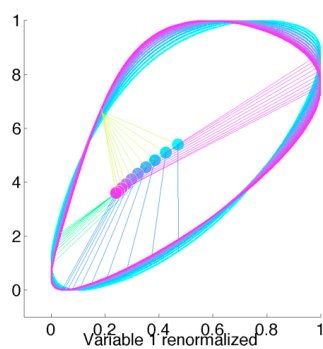**D**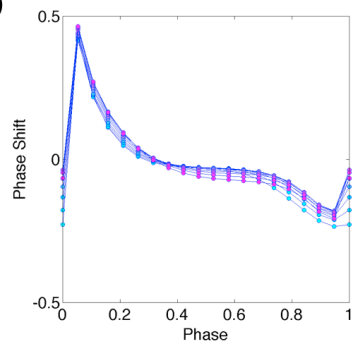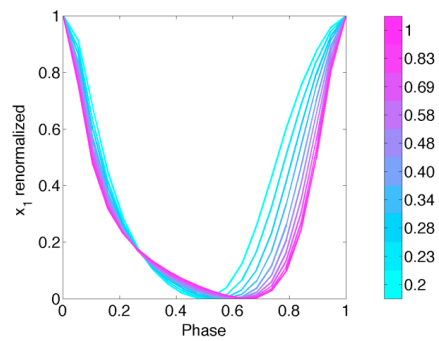

Supplement: Figure S4 — A scaling model evolved with Fitness B. (A) Sketch of the model. (B) Variation of the period as a function of Input. (C) Left : limit cycle for different values of the Input in 1–2 space. Limit cycle varies over one order of magnitude in variable 1 while the period relatively changes of at most Right: Rescaling of those limit cycle to the unit interval for each variable. The orbits overlap again. Circles mark the fixed point (D) Left : Scaling of the PRC was computed by adding a degradation term of for variable 2 for of the period. (Right) Variable 1 as a function of phase for the limit cycles at different temperature. Maximum of 1 is defined as phase for the PRC. (PDF) [file pcbi.1002585.s004.pdf]
