# Supplementary material for: Adaptive Temperature Compensation in Circadian Oscillations
Source: PLoS Comput Biol. 2012 Jul 12;8(7):e1002585. doi: 10.1371/journal.pcbi.1002585 (PMC3395600; doi:10.1371/journal.pcbi.1002585)
Supplement: Table S1 — Detailed properties of limit cycle of network from Fig. 4 for different Input values. (PDF) [file pcbi.1002585.s005.pdf]

Input Value	Min-Max Value for 1	Relative steady state value for 1	Relative period
0.4	0.58-1.5	1.0	1
0.47	0.44- 1.4	0.84	1
0.54	0.33-1.2	0.71	1
0.64	0.26-1.1	0.6	0.99
0.74	0.20-0.98	0.51	0.98
0.86	0.16-0.87	0.43	0.96
1.01	0.12-0.77	0.38	0.94
1.18	0.1-0.67	0.31	0.92
1.37	0.08-0.58	0.27	0.9
1.6	0.07-0.5	0.22	0.86
