# Supplementary material for: Adaptive Temperature Compensation in Circadian Oscillations
Source: PLoS Comput Biol. 2012 Jul 12;8(7):e1002585. doi: 10.1371/journal.pcbi.1002585 (PMC3395600; doi:10.1371/journal.pcbi.1002585)
Supplement: Text S1 — Summary of computational evolution algorithm, detailed properties of network of Fig. 4 , analytic derivation for the adaptive MFL network and equations for the different networks. (PDF) [file pcbi.1002585.s006.pdf]

Supplementary Text S1

Paul François, Nicolas Despierre, Eric Siggia

1 Computational evolution

1.1 Evolutionary dynamics

Genetic networks are evolved by repeated rounds of selection, growth and mutation. Typically 40 networks are followed in parallel. At each step of the algorithm, equations corresponding to the networks are integrated, and a " fitness" or " scoring" function is computed. Networks are then ranked according to this fitness; the best half is retained, then each network kept is copied and mutated. Mutations are of two types : mutations changing the kinetic constants within the networks or mutations changing the topology of the network, i.e. adding/removing new proteins or interactions. At each generation, a given mutation has a pre-determined probability to happen. Mutations changing kinetic parameters in the network are assumed to have a higher probability of occurrence than mutations changing network topologies, and among the latter, the probability of removing existing interactions is higher than probability of adding new interactions. This choice of probabilities corresponds to a biologically realistic limit for which the most probable evolutionary event is to modify existing interactions, the second most probable event being to delete interactions and creating new ones is the least probable. Results of evolution described in this chapter are largely independent of the precise choice of mutation rates as long as simulations are run in this limit.

After the mutation step, the entire process is iterated. A "generation" is one iteration of this selection/growth/mutation process, and corresponds to many generations in a real organism since we are only concerned with mutations in the one network under study. This procedure favours the evolution with time of network topologies and parameters satisfying the fitness function.

1.2 Fitnesses

Mathematical details of the fitnesses are given in the main text, we schematically summarize them in the following.

1.2.1 Fitness A

Variables (ie genes) in the network are designated as an Input and an Output. The dynamics of the Input is completely prescribed and the fitness is computed from the behavior of the Output. For each network, we want to select for both entrainment and temperature compensation. To do so, we define a time interval (typically 12 periods of the desired period of the oscillator) and simulate network with different Input dynamics :

- we first simulate a network with an oscillating Input over the total time window (dynamic 0). We further include several random phase shifts of the Input dynamics during the first cycles.

- then we simulate a network with an oscillating Input identical to dynamic 0 for the first third of the window, then exponentially decaying towards n different constant values between 0 and 2 (dynamic 1 to n)

Fitness is then computed as follows: for dynamic 0, we compute the absolute value of the correlation between Input and Output. Then for dynamics 1 to n, we compute the correlation of the Output for dynamics i with the Output for dynamics 0. We averaged all these correlations to compute the fitness. If all these correlations are maximum, it means that there is perfect entrainment (because the random phase shifts disfavor simple memory of the phase) and period compensation (because dynamics 1 to n impose oscillations similar to dynamic 0 for constant Inputs of varying levels). This fitness was used to evolve the networks displayed in Fig. 4.

1.2.2 Fitness B

We used an other scheme to relax the assumption that Output shape should be strongly correlated to Input shape :

- we kept dynamics 0 as defined.

- then we simulate a network with an oscillating Input identical to dynamic 0 for the first third of the window, then suddenly freezing it to n different constant values between 0 and 2 (dynamic 1 to n)

We still compute absolute value of the correlation between Input and Output for dynamics 0, but for dynamics 1 to n, we count the number of peaks of the Output during the time window where Input is constant, and compute the relative difference between the number of these peaks and the number of peaks of the Input signal for dynamics 0. We add this term to the correlation for dynamics 0. This imposes that the network is entrained but constrains much less the shape of the free running limit cycle.

1.3 Code bloating and pruning

One characteristic of evolutionary simulations is the phenomenon of " code-bloat" : "core" working networks are often embedded into bigger ones due to past evolutionary history but without any functional roles. To identify the most parcimonious sub-network accounting for a function, we use a pruning evolutionary procedure : once a working network topology has been identified, we run our evolutionary simulations in a mode were nodes are randomly pruned and only networks keeping a constant fitness are selected. All networks displayed in this chapter represent such core networks.

1.4 Numerical implementations

The latest version of our algorithm is implemented in Python, one of the most convenient language for systems biology [2]. Networks are defined as bipartite graph, connecting "species" (genes and proteins) to Interactions (transcription, transcriptional regulations, protein-protein interactions, ...). Networks are encoded using the Networkx package [3] and customized classes defining parameters for any interaction. The evolution algorithm per se (including growth and mutations) is encoded in Python. However, Python itself appeared too slow to actually integrate differential equations necessary to fitness computation for each network, so a Python to C dynamical interpreter has been designed, which takes as an input a network in Python and then generates a compiled C code used to numerically compute the network behaviour. Equations are generally integrated using an Euler algorithm, with a time-step chosen a priori to be much smaller than any typical time-scale that could appear in the system. This fitness computation process can be naturally parallelized.

2 More detailed properties of Network of Fig. 4

2.1 Relative period and fixed point value of network of Fig.4 for different value

Table 1 gives the relative period and fixed point value for variable 1 for different values of Input for network shown on Fig. 4.

2.2 Variability of Phase Resetting Curves

Figure S 1 presents PRCs of network of Fig. 4 for various perturbations and different values. Interestingly the PRCs have the same shape and actually overlap for most of these perturbations for different Input values.

2.3 Simulation of mutants for network of Fig. 4

Figure S2 represents the relative period of the oscillator of Fig.4 for different "mutants" simulating 2-fold individual variations of parameters. Interestingly, most parameters have very little influence on the relative variation of the period with the Input, while they can actually modify quite significantly the period itself. This suggests that period compensation is a "structural" property of the network, relatively independent of the precise value of the parameters. The most significant parameters influencing values of period are degradation rates of species 2 and 3 (resp. dark and light blue on Fig.S2). The most significant parameter perturbing period compensation is the coupling between Input and species 1 (yellow parameter).

3 Dynamics of the Adaptive Mixed Feedback Loop

Following [4] we consider the following Mixed Feedback Loop model (MFL) :

$$\begin{aligned} \dot{g} &= \theta(1-g) - \alpha g A && (1) \\ \dot{r} &= f(I)\rho_r(1-g) - \delta_r r && (2) \\ \dot{B} &= \beta r - \gamma AB - \delta_B B && (3) \\ \dot{A} &= f(I)\rho_A - \gamma AB - \delta_A A && (4) \end{aligned}$$

where A, B are proteins and g models the activity of the promoter of gene b whose message level is r. We assume that production rates of A and B depend on the Input in a similar way via the term $f(I)$. The production of B is decomposed into several steps to provide a delay in the negative feedback on A. There would be no qualitative change in the properties of the model if similar terms were included for A. For simulations, we use $\theta = 0.04, \alpha = 0.001, \rho_r = 5, \delta_r = 0.02, \rho_A = 100, \gamma = 1, \beta = 3, \delta_B = 0.1, f(I) = I, \delta_A = 0.01$.

3.1 Adaptive steady state

The crucial assumption for adaptation is to assume that degradation δ_A of A is very small (i.e. negligible relative to the titration rate γB), as found by computational evolution in [1]. At steady state, we then have :

$$\begin{aligned} g &= \frac{\theta}{\theta + \alpha A} = g^*(A) && (5) \\ r &= f(I)\frac{\rho_r(1-g^*(A))}{\delta_r} = f(I)r^*(A) && (6) \\ B &= \frac{\beta f(I)\beta r^*(A)}{\gamma A + \delta_B} = f(I)B^*(A) && (7) \\ 0 &= f(I)\rho_A - \gamma A f(I)B^*(A) = f(I)(\rho_A - B^*(A)) && (8) \end{aligned}$$

where $g^*(A), r^*(A), B^*(A)$ are pure functions of A and other parameters than Input I) so that for A, we get, at steady state :

$$B^*(A) = \rho_A \quad (9)$$

This implicit equations defines steady state for A, which is then independent of the Input, demonstrating adaptation of the steady state with respect to the Input. This can be seen on Figure 5 Panel C of the main paper.

3.2 Scaling of the limit cycle and period independence

Because of the titration γAB, proteins A and B can not coexist in this model at the same time. A consequence is that the MFL limit cycle alternates between

two phases : a Phase I where $A >> B$ and a Phase II where $B >> A$. Complete study of the limit cycle is presented in [4] and a summary of the argument is detailed below. During Phase I, A is big and we can actually adiabatically eliminate B and g which are very small, so that we find

$$\begin{aligned} \dot{r} &= f(I)\rho_r - \delta_r r & (10) \\ \dot{A} &= f(I)\rho_A - \beta r - \delta_A A & (11) \end{aligned}$$

(again, in that phase, δ_B is negligible relative to γA). During Phase II, B is big and we can eliminate A which is negligible to get :

$$\begin{aligned} \dot{g} &= \theta(1-g) & (12) \\ \dot{r} &= f(I)\rho_r(1-g) - \delta_r r & (13) \\ \dot{B} &= \beta r - f(I)\rho_A - \delta_B B & (14) \end{aligned}$$

We proceed to rescale $X = r, A, B$ by the function $f(I)$: calling $\tilde{X} = \frac{X}{f(I)}$ we immediately find for Phase I

$$\begin{aligned} \dot{\tilde{r}} &= \rho_r - \delta_r \tilde{r} & (15) \\ \dot{\tilde{A}} &= \rho_A - \beta\tilde{r} - \delta_A \tilde{A} & (16) \end{aligned}$$

and for Phase II :

$$\begin{aligned} \dot{g} &= \theta(1-g) & (17) \\ \dot{\tilde{r}} &= \rho_r(1-g) - \delta_r \tilde{r} & (18) \\ \dot{\tilde{B}} &= \beta\tilde{r} - \rho_A - \delta_B \tilde{B} & (19) \end{aligned}$$

We have therefore completely absorbed the Input influence in the rescaling ; neglecting the short transition zone between the two phases (which contributes to the period only at higher order in $1/\sqrt{\rho_A \gamma}$, very small compared to the typical period [4]), this analysis implies that, in a very similar way to the Goodwin model as described in the main text :

- the limit cycle scales as $f(I)$
- the period is independent of I
- any multiplicative PRC is independent of I

We further check that the MFL network can be entrained by I (data not shown).

4 Other examples of evolved networks

Many solutions were easily found by the evolutionary scheme described in previous sections. We picked up two other examples of networks evolved using this algorithm, with associated scaled limit cycles and PRCs. Figure S3 gives another example of network evolved with Fitness A. Figure S4 gives another example of network evolved with Fitness B. Both networks have wide variation of the limit cycle for at least one variable, period compensation, orbit scaling, and associated PRC invariance. Thus Fitness B sometimes results in networks with the scaling properties that were explicitly selected for under Fitness A.

5 Network equations

We provide MATLAB files containing ODEs for all networks presented in this paper. The Input variable is the last argument of the functions.

Network of Fig.4

```
function ds=Fig4(~,s,s0)

%Function defining time derivatives for network of Fig. 4.
%Uses Utilities HillA and HillR, s0 is the Input


ds=zeros(4,1);


%degradation rates

ds(1)=-0.049678*s(1);

ds(2)=-0.629237*s(2);

ds(3)=-0.533065*s(3);

ds(4)=-0.998394*s(4);




ds(1)= ds(1)+ 0.999444*HillR(s(3),0.196395,4.782197);

ds(3)= ds(3)+ 0.979918*HillA(s(2),0.367591,4.776183);

ds(2)= ds(2)+ 0.631913*s0*s(1)-0.214075*s(2);


ds(1)= ds(1)+ -0.631913*s0*s(1)+0.214075*s(2);

ds(4)= ds(4)+ 0.728435*s(1)*s(3)-0.174149*s(4);

ds(1)= ds(1)+ -0.728435*s(1)*s(3)+0.174149*s(4);

ds(3)= ds(3)+ -0.728435*s(1)*s(3)+0.174149*s(4);
```

Network of Fig.6

```
function odefun=Fig6(~,x,s0)


%Function defining time derivatives for network of Fig. 6.
%Uses Utilities HillA and HillR, s0 is the Input



odefun=zeros(7,1);


odefun(1)=-0.616633*x(1)+0.997857*HillR(x(4),0.014614,4.635085)*HillR(↩
    x(7),0.441944,1.636007)-0.128611*s0*x(1)+0.638779*x(2);

odefun(2)=-0.017157*x(2)+0.128611*s0*x(1)-0.638779*x(2);
odefun(3)=-0.568141*x(3)+0.735827*max(HillA(x(2),0.443434,4.906333),↩
    HillA(x(7),0.184899,3.123139))-0.355762*s0*x(3)+0.707227*x(4);

odefun(4)=-0.340442*x(4)+0.355762*s0*x(3)-0.707227*x(4);
odefun(5)=-0.178738*x(5)+0.506883*max(HillA(x(1),0.784509,3.597002),↩
    HillA(x(6),0.388452,0.925010))-0.860478*s0*x(5)+0.245151*x(6)↩
    -2*0.924989*x(5)*x(5)+2*0.546817*x(7);
odefun(6)=-0.618530*x(6)+0.860478*s0*x(5)-0.245151*x(6);
odefun(7)=-0.882519*x(7)+0.924989*x(5)*x(5)-0.546817*x(7);
```

Network of Fig. S3

```
function ds=FigS3(~,s,s0)

%Function defining time derivatives for network of Fig. S3
%Uses Utilities HillA and HillR, s0 is the Input

ds=zeros(6,1);

ds(1)=-0.017242*s(1);
ds(2)=-0.682375*s(2);
ds(3)=-0.274146*s(3);
ds(4)=-0.877576*s(4);
ds(5)=-0.725935*s(5);
ds(6)=-0.327922*s(6);

rate=0.999444*HillR(s(4),0.077706,3.873770)*HillR(s(5)↩
    ,0.167392,4.960077);
ds(1)=ds(1)+rate;

rate=0.844962*HillA(s(2),0.462754,4.936277)*HillR(s(6)↩
    ,0.303372,3.401030);
ds(3)=ds(3)+rate;

rate=0.980526*HillA(s(4),0.245517,2.393060);

ds(5)=ds(5)+rate;

rate=0.937099*s0*s(1)-0.969777*s(2);


ds(1)=ds(1)-rate;
ds(2)=ds(2)+rate;
```

```
rate=0.994958*s(1)*s(3)-0.084167*s(4);

ds(1)=ds(1)-rate;
ds(3)=ds(3)-rate;
ds(4)=ds(4)+rate;


rate=0.969937*s(1)*s(5)-0.175595*s(6);
ds(1)=ds(1)-rate;
ds(5)=ds(5)-rate;
ds(6)=ds(6)+rate;
```

Network of Fig. S4

```
function odefun=FigS4(~,x,s0)

%Function defining time derivatives for network of Fig. S4
%Uses Utilities HillA and HillR, s0 is the Input



odefun=zeros(5,1);



odefun(1)=-0.765729*x(1)+0.969707*HillR(x(4),0.032403,3.957494)↩
    -0.847064*s0*x(1)+0.534346*x(2);
odefun(2)=-0.071591*x(2)+0.847064*s0*x(1)-0.534346*x(2);
odefun(3)=-0.050645*x(3)+0.877848*HillA(x(2),0.245479,4.965232)↩
    -2*0.717585*x(3)*x(3)+2*0.058512*x(4)-0.313311*s0*x(3)+0.999867*x↩
    (5);
odefun(4)=-0.751772*x(4)+0.717585*x(3)*x(3)-0.058512*x(4);
odefun(5)=-0.110169*x(5)+0.313311*s0*x(3)-0.999867*x(5);
```

Utilities

```
function a=HillA(x,y,n)
b=exp(n*log(x/y));
a=b/(1+b);
```

```
function a=HillR(x,y,n)
b=exp(n*log(x/y));
a=1/(1+b);
```

References

[1] Paul Francois and Eric D Siggia. A case study of evolutionary computation of biochemical adaptation. *Physical Biology*, 5(2):26009, 2008.

Table 1: Detailed properties of limit cycle of network from Figure 4 for different Input values

Input Value	Min-Max Value for 1	Relative steady state value for 1	Relative period
0.4	0.58-1.5	1.0	1
0.47	0.44- 1.4	0.84	1
0.54	0.33-1.2	0.71	1
0.64	0.26-1.1	0.6	0.99
0.74	0.20-0.98	0.51	0.98
0.86	0.16-0.87	0.43	0.96
1.01	0.12-0.77	0.38	0.94
1.18	0.1-0.67	0.31	0.92
1.37	0.08-0.58	0.27	0.9
1.6	0.07-0.5	0.22	0.86

[2] CR Myers, RN Gutenkunst, and James P Sethna. Python unleashed on systems biology. *Computing in Science & Engineering*, pages 34–37, 2007.

[3] http://networkx.lanl.gov/.

[4] Paul Francois and Vincent Hakim. Core genetic module: the mixed feedback loop. *Physical review E, Statistical, nonlinear, and soft matter physics*, 72(3 Pt 1):031908, September 2005.

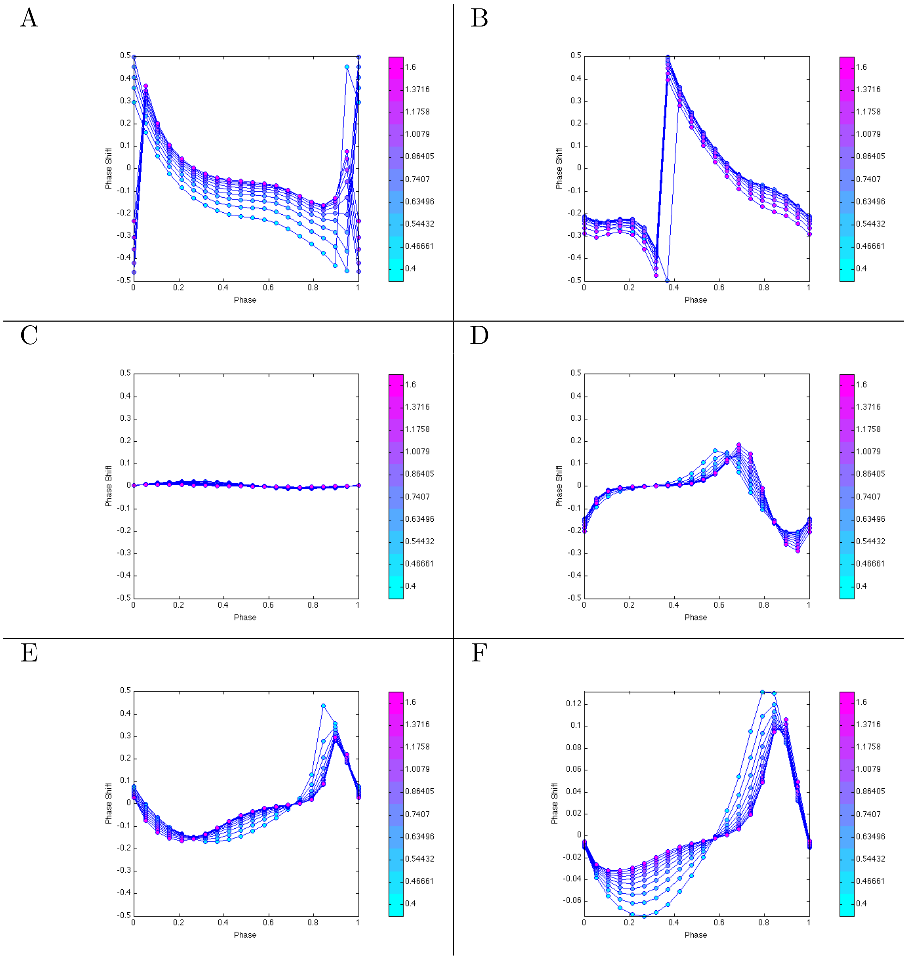

Figure S 1: **Families of PRCs for model of Fig.4** All PRCs are computed by imposing a perturbation for 10 % of the period of a cycle. (A) Strong degradation of species 1 (as described in main text) (B) Strong degradation of species 3 (C) Strong degradation of species 4 (D) 4x increase of transcription of gene 1 (E) 4x increase of transcription of gene 3 (F) 50% relative increase of Input. The strongest departure from input (temperature) invariance is in A since adding degradation to species 1 breaks the adaptation in the initial adaptive system composed of species 0,1,2 in Fig. 3

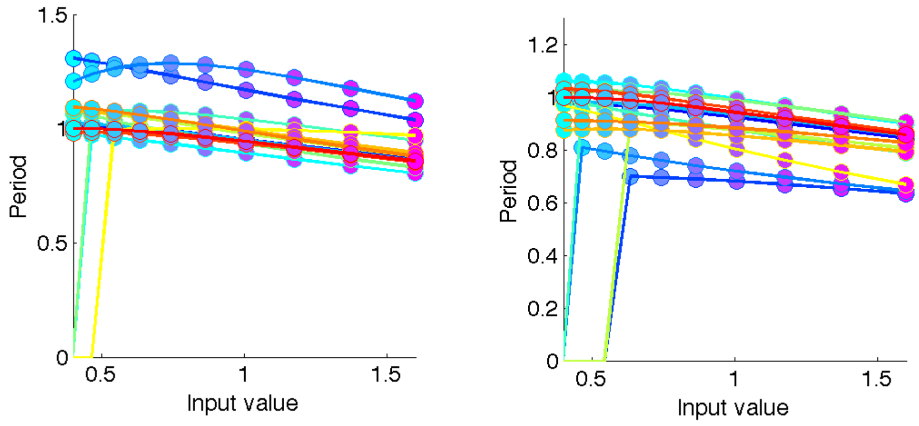

Figure S2: **Relative period as a function of Input for simulated mutants of networks in Fig. 4** Period 0 corresponds to absence of oscillation, periods are computed relative to the original network for the reference Input value of 0.4. Individual period for each mutant can be different from the original network, but for most mutants taken individually, period variation is comparable to the original network. (Left) Parameters individually divided by 2. The most significant relative difference (20 %) is for the parameter in dark blue which corresponds to the degradation rate of the Output (species 2). (Right) Parameters individually multiplied by 2. The most significant relative difference (30 %) is for the parameter in yellow which corresponds to the coupling between the input and the network, so effectively multiplies the input range by 2.

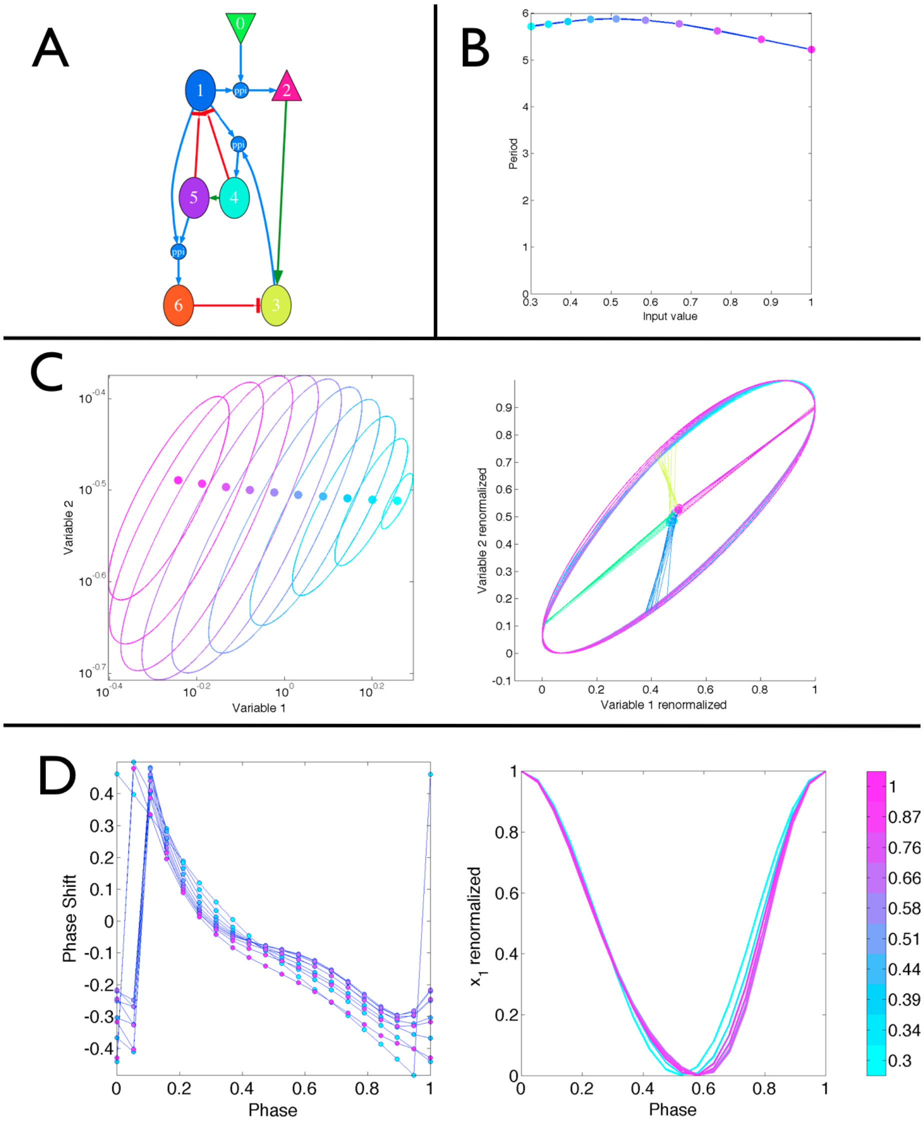

Figure S 3: **A scaling model evolved with Fitness A** (A) Sketch of the model. (B) Variation of the period as a function of Input. (C) Left : limit cycle for different values of the Input in 1-2 space. Limit cycle varies by a factor 4 for variable while the period changes by at most 11% Right: Rescaling of the limit cycles to the unit interval for each variable. The orbits again collapse well. Circles mark the fixed point (D) Left : The PRC was computed by adding a degradation term of $-10s_2$ for variable 2 for 10% of the period. (Right) Variable 1 as a function of phase for the limit cycles at different temperatures. Maximum of 1 is defined as phase 0 for the PRC.

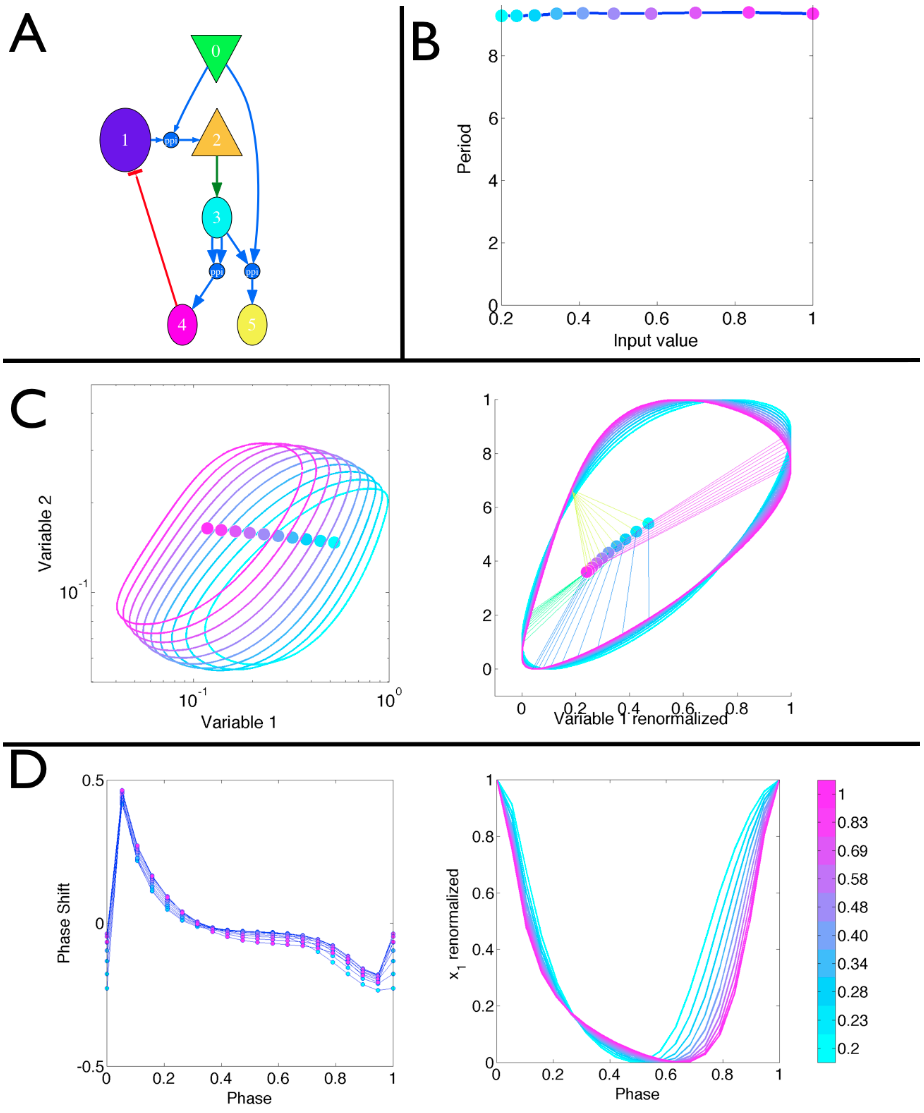

Figure S 4: **A scaling model evolved with Fitness B** (A) Sketch of the model. (B) Variation of the period as a function of Input. (C) Left : limit cycle for different values of the Input in 1-2 space. Limit cycle varies over one order of magnitude in variable 1 while the period relatively changes of at most 1% Right: Rescaling of those limit cycle to the unit interval for each variable. The orbits overlap again. Circles mark the fixed point (D) Left : Scaling of the PRC was computed by adding a degradation term of $-10s_2$ for variable 2 for 10% of the period. (Right) Variable 1 as a function of phase for the limit cycles at different temperature. Maximum of 1 is defined as phase 0 for the PRC.
